# Protocol for a randomized controlled trial of a specialized health coaching intervention to prevent excessive gestational weight gain and postpartum weight retention in women: the HIPP study

**DOI:** 10.1186/1471-2458-12-78

**Published:** 2012-01-25

**Authors:** Helen Skouteris, Marita McCabe, Jeannette Milgrom, Bridie Kent, Lauren J Bruce, Cathrine Mihalopoulos, Sharon J Herring, Malcolm Barnett, Denise Patterson, Glyn Teale, Janette Gale

**Affiliations:** 1 School of Psychology, Faculty of Health, Deakin University, Melbourne, Australia; 2 Department of Psychology, School of Behavioral Science, University of Melbourne, Melbourne, Australia; 3 School of Nursing and Midwifery, Faculty of Health, Deakin University, Melbourne, Australia; 4 Public Health Cluster, Faculty of Health, Deakin University, Melbourne, Australia; 5 Center for Obesity Research and Education, Temple University, Philadelphia, PA, USA; 6 Obstetrics and Gynecology, Eastern Health, Melbourne, Australia; 7 Women and Children's Services, Box Hill Hospital, Eastern Health, Melbourne, Australia; 8 Obstetrics and Gynecology, Sunshine Hospital, Western Health and the University of Melbourne, Melbourne, Australia; 9 Health Coaching Australia, Kangaroo Valley, New South Wales, Australia

**Keywords:** Pregnancy, Postpartum, Gestational weight gain, Weight retention, Health coaching, Randomized controlled trial

## Abstract

**Background:**

Pregnancy is a time of significant physiological and physical change for women. In particular, it is a time at which many women are at risk of gaining excessive weight. We describe the rationale and methods of the Health in Pregnancy and Post-birth (HIPP) Study, a study which aims primarily to determine the effectiveness of a specialized health coaching (HC) intervention during pregnancy, compared to education alone, in preventing excessive gestational weight gain and postpartum weight retention 12 months post birth. A secondary aim of this study is to evaluate the mechanisms by which our HC intervention impacts on weight management both during pregnancy and post birth.

**Methods/Design:**

The randomized controlled trial will be conducted with 220 women who have a BMI > 18.5 (American IOM cut-off for normal weight), are 18 years of age or older, English speaking, no history of disordered eating or diabetes and are less than 18 weeks gestation at recruitment. Women will be randomly allocated to either a specialized HC intervention group or an Education Alone group. Our specialized HC intervention has two components: (1) one-on-one sessions with a Health Coach, and (2) two by two hour educational group sessions led by a Health Coach. Women in the Education Alone group will receive two by two hour educational group sessions with no HC components. Body Mass Index, waist circumference, and psychological factors including motivation, readiness to change, symptoms of depression and anxiety, and body dissatisfaction will be assessed at baseline (14-16 weeks gestation), and again at follow-up: 32 weeks gestation, 6 weeks, 6 months and 12 months postpartum.

**Discussion:**

Our study responds to the urgent need to design effective interventions in pregnancy to prevent excessive gestational weight gain and postpartum weight retention. Our pregnancy HC intervention is novel and innovative and has been designed to be easily adopted by health professionals who work with pregnant women, such as obstetricians, midwives, allied health professionals and health psychologists.

**Trial registration:**

Australian New Zealand Clinical Trials Registry ACTRN12611000331932

## Background

The importance of childbearing in the development of obesity in women has been recognized for over a decade [1]. Pregnancy is a time of significant physiological and physical change for women. In particular, it is a time at which many women are at risk of gaining excessive weight and hence a time where interventions to address overweight/obesity in women should be conducted [2].

### Excessive weight gain during pregnancy

Excessive weight gain in pregnancy is a common health-related problem in Western countries [3]. Women who gain excessive weight during pregnancy have an increased risk of postpartum obesity in themselves and their children [2,4]. Furthermore, 20% of women retain at least 5 kg of gestational weight gain at 6-18 months post birth [5,6], and this weight retention is a strong predictor of maternal overweight and obesity a decade or more after birth [7].

In 2009 the United States (US) Institute of Medicine (IOM) published revised guidelines for how much weight a woman should gain during pregnancy and highlighted the importance of intervention in pregnancy to prevent both postpartum weight retention and childhood obesity [8]. The US IOM recommends that women with a normal weight (Body Mass Index, BMI, of 18.5-24.9 kg/m^2^) should gain between 11-16 kg during their pregnancy; women who are overweight (BMI of 25-29.9 kg/m^2^) should gain 7-11 kg, and obese women (BMI of 30 kg/m^2 ^and above) should gain between 5 to 9 kg [8]. Women who are underweight (BMI less than 18.5 kg/m^2^) should gain between 13 to 18 kg during pregnancy [8]. Excessive gestational weight gain (GWG) is defined as weight gain above these recommended guidelines. The specific cost of excessive gestational weight gain is related to ensuing maternal health problems and fetal outcomes [9], such as preeclampsia [10], maternal hyperglycemia [11], complications with labor/delivery [12,13], infant macrosomia [12,13], late fetal death [14], birth defects [15], and an increased risk of caesarean [10].

### Determinants and correlates of gestational weight gain

The biological and behavioral determinants of excessive GWG include high pre-pregnancy BMI, primiparity, advanced maternal age, higher energy intake, a reduction in physical activity, and lack of advice in relation to recommended guidelines for weight gain during pregnancy [9,16]. The psychological correlates of excessive gestational weight gain include higher depressive [17] and anxiety symptoms [18,19], lower self-esteem [18], misperceived pre-pregnancy body size [17], and greater body image dissatisfaction [20]. Furthermore, it has been shown that greater gestational body image dissatisfaction is associated with higher depressive symptoms in pregnancy (*r*s range from .23-.46) [21-24] and in the postpartum (*r*s range from .26-.54) [24,25] and with higher BMI during pregnancy [20-23]. Therefore, managing weight gain during pregnancy might be more effective if it includes the management of psychological factors, such as body image concerns and depressive symptoms.

### Interventions designed to prevent excessive weight gain during pregnancy: a systematic review of the literature

We conducted a systematic review of interventions designed to prevent excessive GWG in pregnant women across all BMI categories [26]. Our search, which was limited to English papers published between January 1999 and January 2010, revealed 10 intervention studies; three of these studies were conducted in the US [27-29], two in Canada [30,31] and one in Sweden [32,33], Finland [5], Denmark [34], Australia [35], and Belgium [36]. Only one of these studies tracked women from early pregnancy until 12 months post birth [28]; the other studies did not evaluate the effect of the intervention on weight retention in the postpartum, with the exception of Wolff et al., Gray-Donald et al., and Polley et al., who followed women through pregnancy to 4 weeks, 6 weeks, and approximately 8 weeks post birth, respectively [27,28,34]. In the published studies, excessive gestational weight gain was prevented only in: normal-weight women [27], low-income women [28], obese women [33,34], overweight women [35], or not at all [5,30,31,37]. These interventions have focused primarily on behavioral changes in relation to physical activity and/or to eating. Some research findings emphasize the importance of targeting both behavioral and psychological factors in order to maintain weight loss postpartum [20,21,24,25]. In her review of interventions to manage excessive gestational weight gain, Walker also concluded that interventions to date had limited success possibly because psychological factors were not considered [37]. That is, interventions have not included dedicated behavior-change assistance aimed at identifying and addressing behavioral, emotional, cognitive, and situational barriers that might impede behavior change; the lack of consideration of psychological factors was also identified by the US IOM [8].

Since January 2010, seven additional intervention studies have been published [38-44]. The weight status of women targeted (BMI category) and the guidelines used to determine excessive GWG varied between studies. One intervention referred to guidelines recommended by the Swedish Births Registry (weight gain of 6 kg or less for all women) [39] while another did not refer to GWG guidelines at all [38]. A majority of studies included nutritional and/or physical activity behavior components as part of the intervention program [38-42,44] and four of the six interventions included a psychological component in the form of counseling or support [38,39,41,43]. A majority of the seven studies found positive effects of the intervention on the restriction of GWG [39,41-43] but results were obtained for women in different BMI categories. For example, GWG was restricted amongst normal weight women but not overweight or obese women [43], in obese women with a BMI greater than 35 kg/m^2 ^(compared to women with a BMI between 30 kg/m^2 ^and 35 kg/m^2^) [39] and in less than half of overweight or obese women (31%) [42]. Further, one study reclassified obese women (BMI > 30 kg/m^2^) as overweight and found no effect of an exercise intervention on GWG, when comparing normal/overweight women to controls using intention-to-treat analyses [44]. Only one adequately powered randomized controlled trial (RCT) [43] examined the effectiveness of an intervention to reduce excessive GWG among normal weight, overweight and obese women. Significant effects were not found for the intervention in restricting GWG in overweight or obese women according to US IOM (1990) guidelines [45], however significant effects were found for normal weight women. Further, a greater percentage of women across all weight categories reached at or below their preconception weight six months after the birth of their baby [43].

The recent literature reflects the findings of the review by Skouteris et al. [26] that GWG was reduced only in some populations, and often not according to those levels recommended by the US IOM [8,45]. Furthermore, similar limitations to those present in previously reviewed interventions continue to be evident. The effect of the intervention on postpartum weight retention was not evaluated in three studies [38,39,41], and in one study intervention women were compared to an historical control group [40] and another did not refer to a control group at all [39]. Importantly, none of the recent interventions incorporated psychologically-based components of behavior change: only targeting behavior change associated with diet and physical activity. The current study addresses this gap.

### A specialized health coaching intervention

In recent times the emphasis on using a combination of psychological and behavioral interventions in addition to patient education has been stressed, given the growing recognition that information/education/advice alone is not sufficient to produce significant changes in health behavior [46] and that adoption of new behaviors is more likely if patients are encouraged to be involved in behavioral intention at the time of being provided with health information [47]. Indeed, a recent study showed, with a large cohort of non-pregnant women, that only a very small proportion of women planning a pregnancy followed the recommendations for nutrition and healthy lifestyle, when this was given as advice alone [48].

Health Coaching (HC) is one strategy that is likely to engage women in the behavior change necessary to prevent excessive weight gain during pregnancy [49,50]. HC provides individual, one-on-one interventions (face to face, telephonic, internet or multimodality), and the principles can also be used in small group education situations to help participants to integrate new knowledge into their personal behavior change plans. The result is to create an immediate intention to act, and increases the likelihood of behavior change [47]. The major tasks for the HC health professional are to: (1) assist the client to engage in behavior change for self-motivating reasons; (2) increase the client's chances of successful and lasting behavior change once he/she has decided to make particular changes; and (3) provide health information and education and to correct misinformation as required, in a way that increases adherence and avoids creating resistance to change. The results of the only study evaluating this approach among 56 obese pregnant women (*n = *56) found that the majority of women made positive changes to their eating and exercise habits during pregnancy, and just under half of the women gained within the US IOM [8] recommended weight gain during pregnancy of about 5 to 9 kg; all of the women also stated they would recommend the program to a friend [32]. Whether these women were able to maintain their change in attitude, eating habits and weight control in the postpartum was not investigated.

The Health Coaching Australia (HCA) Model [49] is an integrated Health Coaching model drawing on evidence-based psychological theories, practices and principles, such as Social Cognitive Theory [51], Readiness to Change, Importance, and Confidence [52], the Transtheoretical Model [53], and the Theory of Planned Behavior [54]. The HCA Model offers a unique intervention approach for preventing excessive gestational weight gain and weight retention in the postpartum because it provides behavioral, cognitive, situational, and emotional change facilitation: not just behavioral change facilitation [49].

We have developed a novel pregnancy HC intervention that has two components. (1) One-on-one sessions with a Health Coach that a) promote women's adoption of healthy lifestyle behaviors for the purpose of weight management, and b) address mood management and body image issues that commonly arise during pregnancy. (2) Educational group sessions, that augment the one-on-one sessions; the aim is to provide new mothers with additional information related to healthy behaviors and mood, and to support and assist them in initiating, maintaining, and achieving their goals for healthy behavior change.

### Specific aim 1

To compare the effectiveness of a specialized HC intervention during pregnancy, in preventing excessive gestational weight gain and postpartum weight retention 12 months post birth, compared to education alone. Hypothesis: In comparison to the education alone group, there will be a significantly greater proportion of women in the intervention group who: (1) will gain weight within the recommendations outlined by the American IOM [8] for gestational weight gain; (2) will have returned to their pre-pregnancy weight by 12 months post birth.

### Specific aim 2

To evaluate the mechanisms by which our HC intervention impacts on weight management both during pregnancy and post birth by tracking the relationships among the following variables: (1) shifts in motivation to adopt healthy lifestyle behaviors (including managing mood changes and body image concerns) for the purposes of weight management; (2) confidence in adopting healthy lifestyle behaviors for the purposes of weight management; (3) actual behavior changes (goal behavior initiation, achievement, and maintenance); (4) physiological and psychological outcomes - i.e., Body Mass Index (BMI), waist circumference, depressive symptoms, anxiety, and body image concerns. Hypothesis: It is hypothesized that, in comparison to education alone, the intervention will promote a shift in motivation to adopt, and an improvement in women's confidence and ability to overcome barriers to, healthy eating and physical activity, and better management of mood and body image. These changes are predicted to lead to an initiation of healthy behavior changes for weight management and then to sustained healthy behavior changes, lower levels of depressive and anxiety symptoms, and lower body dissatisfaction.

## Methods/Design

### Overall study design

This study will be a randomized controlled trial (RCT) with pregnant women randomly allocated to either the HC Intervention or an Education Alone control group. The study will be conducted and reported in line with CONSORT recommendations [55]. Baseline assessment of study variables will occur at 14-16 weeks gestation; this allows women to consider the pregnancy certain (since the threat of miscarriage has substantially decreased) and to participate at a standardized time point. Follow up assessments will take place at 32 weeks gestation, and at 6 weeks, and 6 and 12 months postpartum. The study was approved by the Eastern Health Human Research Ethics Committee on the 25th March, 2011 (EH-HREC E40/1011) and Deakin University Human Research Ethics Committee on 21st April, 2011 (DUHREC 2011-087).

### Participants

Participants will be 220 pregnant women. Women will be able to participate if they provide informed consent, have a BMI > 18.5 (US IOM cut-off for normal weight), are 18 years of age or older, English speaking and less than 18 weeks gestation at recruitment with no history of disordered eating or diabetes. After baseline assessment, women who meet the eligibility criteria will be randomly allocated to either the HC Intervention group (*n *= 110) or the Education Alone group (*n *= 110). A coded, double-blinded, variable-length permuted blocks randomized treatment allocation schedule, produced by computer algorithm, will be used.

### Recruitment strategies

Women from a diverse range of socioeconomic and ethnic backgrounds will be recruited predominantly from Birralee Maternity Service, Eastern Health, for which University and Hospital ethics approvals have been granted.

### Procedure

#### The intervention

##### The Health Coaching (HC) Intervention

In addition to usual care, women in this group will take part in a HC Intervention program. Our specialized HC Intervention has two components: (1) one-on-one sessions with a Health Coach, and (2) educational group sessions lead by a Health Coach. Each of these components are described in more detail below and presented as a timeline in Table 1. The Health Coach is accredited with Health Coaching Australia and is a practicing Physiotherapist.

**Table 1:** Overview of intervention timeline: Health coaching, education sessions and assessments

	Intervention *n *= 110	Education Alone *n *= 110
**Pregnancy**		

14-16 weeks	First assessment (baseline questionnaire measures), BMI, waist circumference	First assessment (baseline questionnaire measures), BMI, waist circumference

16-18 weeks	First one-on-one session with Health Coach (face to face; 1 hour)	Usual care

20 weeks	First 2 × 2 hour group education session	First 2 × 2 hour group education session

22 weeks	Second 2 × 2 hour group education session	Second 2 × 2 hour group education session

24 weeks	Second one-on-one session with Health Coach (over the phone; 30 minutes)	Usual care

28-30 weeks	Two brief follow-up phone health sessions, (15 minutes each)	Usual care

32 weeks	Second assessment (follow-up questionnaire measures)	Second assessment (follow-up questionnaire measures)

**Postpartum**		

6 weeks	Third assessment (follow-up questionnaire measures)	Third assessment (follow-up questionnaire measures)

6 months	Fourth assessment (follow-up questionnaire measures)	Fourth assessment (follow-up questionnaire measures)

12 month	Fifth assessment (follow-up questionnaire measures), BMI, waist circumference Interviews with 20 women	Fifth assessment (follow-up questionnaire measures), BMI, waist circumference Interviews with 20 women

##### One-on-one sessions

In the one-on-one component of the intervention, a HC will promote patient adoption of healthy lifestyle behaviors (nutrition, physical activity, energy levels for weight management and will address mood and body image issues that commonly arise during pregnancy). Two one-on-one sessions with a trained HC professional will be conducted to address motivational factors, identify potential barriers to adopting a healthy weight-management regime, and assist pregnant women to identify individually tailored solutions to overcome these barriers. The HC will aim to build long-lasting skills about decision making, problem solving, planning and mood management. In these two sessions, the first for one hour at 16-18 weeks gestation (face to face, in a consulting room at the maternity service) and the second for 30 minutes at 24 weeks gestation (via telephone), goals and an action plan will be established collaboratively to initiate health behavior change relevant to healthy weight-management. Readiness, importance and confidence levels will be monitored to ensure that each patient is actively motivated to make the chosen changes and has a reasonable level of confidence in achieving the set goals. The Health Coach will respond appropriately to varying levels of readiness, importance and confidence using well recognized motivational interviewing and solution-focused counseling techniques. These techniques will be augmented by specifically designed techniques as required in order to address cognitive barriers to health behavior change.

##### Follow-up telephonic health coaching sessions

Two brief follow-up telephone health coaching sessions, for 15-minutes each time, will support ongoing behavior change maintenance and progress toward healthy weight-management goals. These telephone sessions will take place anywhere between 28-30 weeks gestation depending on the woman's needs or preferences.

##### HC educational group sessions

In addition to the individual HC sessions, there will be 2 × 2 hour group education sessions that include the following key topics: dietary and physical activity guidelines in pregnancy; beliefs about pregnancy weight gain and body image; beliefs about postpartum weight loss and body image; mood changes during pregnancy; factors that protect a women against elevated depressive symptoms; and the negative consequence of postpartum weight retention for maternal and infant/child health. These group sessions will support behavior change processes begun in the initial individual session, and hence will incorporate HC; they will take place when women are 20 and 22 weeks gestation.

#### Education alone

In addition to usual care, women in this condition will receive 2 × 2 hour education group sessions that provide factual information only; that is, there will be no HC incorporated into these educational sessions and an educator, not the Health Coach, will run these sessions. The educator is a qualified workplace trainer and assessor.

### Measures

Women will meet with researchers at 14-16 weeks gestation to collect demographic information (including pre-pregnancy BMI) and obtain anthropometric measurements (BMI and waist circumference). Women will also be given a copy of the US IOM gestational weight gain guidelines [9] and baseline measures of motivation, importance, confidence and readiness to adopt healthy lifestyle behaviors for weight management, perceived social support, depressive symptoms, body dissatisfaction, dietary habits and physical activity will be collected by questionnaire. Follow-up assessment of measures will take place at 32 weeks gestation, and at 6 weeks, 6 and 12 months post birth, with the exception of anthropometric measures which will be assessed at baseline and at 12 months post birth.

All measures in the questionnaire, with the exception of the first 14-16 week assessment session, will be collected in one of two ways: (1) via self-report questionnaires sent to the home of women with a request to return completed questionnaires (that will take no more than 30 minutes to complete) to the researchers in reply-paid envelopes provided to them; (2) by researcher interview over the phone. Women will be able to choose the method of data collection that suits them best. Anthropometric measures will be obtained in person by trained research staff.

### Primary outcomes

#### BMI

Height will be measured by researchers with a stadiometer at the first and last assessments (14-16 weeks gestation and 12 months postpartum) for both groups. Women will be weighed each time wearing light clothing and no shoes. Pre-pregnancy weight will be based on self-report; weight at 14-16 weeks gestation and 12 months postpartum will be measured by the researchers using electronic scales. Height and weight measurements will be used to calculate BMI (kg/m^2^). At all other assessment time points, women will be encouraged to consult with their healthcare professional to provide additional objective weight measurements (including the last weight reported prior to delivery), and in the postpartum we will ask women to be weighed at their Maternal and Child Health appointment. Women will record these measurements in a 'weight passport' provided by researchers.

Waist circumference will be measured by trained researchers using a standard tape measure when height and weight measurements are obtained.

### Secondary outcomes

All the measures below will be measured at each of the assessment time points noted in Table 1.

#### Readiness to change

A series of 10 questions developed by the researchers assess importance, readiness and confidence of women to change their diet and physical activity habits (i.e. "How important/confident/ready are you right now to take some action to manage your diet/physical activity habits to be healthy, given everything else that is going on in your life right now?"), along with a subjective rating of how healthy their diet and physical activity habits are (i.e. "On a scale of 0-10 please rate how healthy your feel your current diet/physical activity habits are"). Participants are asked to provide a rating on a scale from 0-10 (not at all - extremely) for each item.

#### Motivation to change

Three items developed by the researchers will assess importance, confidence, and motivation to initiate, and if initiated to maintain, healthy behavior changes.

#### General distress and psychopathology

Measures include the short form of the Depression Anxiety Stress Scale and the Edinburgh Postnatal Depression Scale. The Edinburgh Postnatal Depression Scale [56] cut-off scores between 12.5 and 13 result in excellent sensitivity and very good specificity for postpartum use compared to research diagnostic criteria.

#### Body dissatisfaction

The Body Attitudes Questionnaire is a multi-dimensional set of scales developed and validated in Australia; subscales related to body size dissatisfaction: Strength and Fitness, Attractiveness, Salience of Weight and Shape and Feeling Fat [57] will be used. The current and ideal versions of the Contour Drawing Rating Scales [58] will also be administered in the postpartum.

#### Exercise

The Active Australia Survey [59] is a validated questionnaire that measures physical activity behavior in adults, will be used to measure engagement in physical and sedentary activities (duration and nature); these behaviors are retrospectively reported by women for the past 7 days.

#### Food intake

A modified version of the valid [60] and reliable [61] National Nutrition Survey Food Frequency Questionnaire [62] will be used to assess food intake for the last 3 months.

#### Other relevant variables

Other variables will be included to ensure comprehensive assessment and will be examined as secondary, or covariate variables. These include parity, socioeconomic status, substance use, healthcare resource-use, perceived social support, coping style and appraisal of stressful events, beliefs about gestational weight gain, labor experiences (e.g., length, type), breast-feeding experiences, birth weight and delivery date.

### Economic evaluation

This study will include a formal economic evaluation to assess the cost-effectiveness of the intervention. The economic evaluation will comprise a cost-consequences analysis whereby the incremental costs of the intervention will be compared to the full spectrum of outcomes included in the study. This means that a series of cost-effectiveness ratios will be determined rather than just one - such an approach has been shown to be useful to decision-makers. The economic analysis will be primarily from the perspective of the health care sector. The intervention costs will be determined using standard financial records and interviews with project staff and financial officers. The evaluation will first measure and value any change to the use of health care resources over the period of the study between the two arms of the trial and then compare any additional costs to the additional outcomes achieved. Standardized economic evaluation techniques including incremental analysis of mean differences and bootstrapping to determine confidence intervals will be used in the evaluation.

### Qualitative interviews

We will invite 20 women (at 12 months post birth) from the HC Intervention and Education Alone groups (*n *= 40) to take part in an interview to obtain qualitative data in relation to barriers to adopting healthy behaviors for weight management and the support or resources that women found most useful in achieving their gestational weight gain/postpartum weight loss goals.

### Power, sample size, and retention

Given that the primary outcome of the study is maternal BMI, we aim to detect a clinically relevant difference in BMI of 0.8 kg/m^2 ^at 12 months postpartum. This difference is in accordance with the New Life (style) RCT that is currently being conducted in the Netherlands [63], that targets weight advice, weight development, nutrition and physical activity habits, but fails to target motivational variables, behavior-change assistance or psychological factors, such as depressive symptoms, anxiety symptoms, and body image. Consequently, the required *n *to detect this difference with 80% power at *α *= 0.05 and a standard deviation of 2 kg/m^2^, is 110 women per group; we have adopted this SD because we will also be measuring weight objectively at baseline and 12 months postpartum, removing any bias associated with self-report. Allowing for attrition of 20%, we will recruit 264 women and randomly assign 132 women into the HC Intervention and 132 women into the Education Alone control group. Our estimate of a drop-out rate is conservative and based on our previous large longitudinal studies with pregnant women and multiple time points [21,23,64]. As in our previous longitudinal pregnancy studies, this attrition rate of 20% takes into consideration that 5-7% of women may be diagnosed with gestational diabetes, which we expect to occur at 24-26 weeks; these women will therefore not affect the intervention, because we will over sample. A permuted blocks randomized treatment allocation schedule, produced by computer algorithm, will be used for the randomization process. To facilitate retention, we will build rapport with the participants through a study website, a regular newsletter, telephone support and advanced notification of assessments.

### Analyses

We will adhere to 'intention-to-treat' principles following CONSORT Statement guidelines to prevent introduction of systematic bias [48]. As such, baseline data will be secured prior to treatment allocation, missing values will be scrutinized to check for non-random distribution and primary analyses will be executed twice: once using observed data, and once using multiple imputation under multivariate normal assumptions using methods given by Schaffer [65], so that all participants will be analyzed in their allocated condition. Analyses of covariance (with "other relevant variables" from above as covariates) will test the between group differences in the primary and secondary outcomes at each assessment time point and across time points. The confounder-adjusted analysis of the proportion of women returning to pre-pregnancy weight at 12 months post birth in each group will be tested using a logistic regression model. The second hypothesis will be tested using hierarchical regressions and path analysis. In addition, to further clarify the direction of effects between two variables across multiple time points, cross-lagged panel analyses will be conducted, e.g., to determine whether the level of maternal activity/depressive symptoms is a cause or consequence of higher BMI. Finally, the interview transcripts will be analyzed using elements of phenomenology and thematic content analysis to determine barriers to adopting healthy behaviors for weight management and the support or resources that women find most useful in achieving their gestational weight gain/postpartum weight loss goals [25,66].

## Discussion

With approximately 50% of Australian adult women of childbearing age being overweight or obese [67], the impact of pregnancy on women's weight status cannot be ignored [37]. Despite the puerperal period being a time of increased risk for both mother and child, it is also a time of unique opportunity, since pregnant women have frequent interactions with the health care system and may be especially receptive to behavior change recommendations, as evidenced by campaigns for smoking cessation in pregnancy [20]. Promoting healthy weight management around a given pregnancy is likely to favorably influence a woman's health entering her succeeding pregnancies, thus improving the health of not only her index pregnancy, but also subsequent children [68].

Our study responds to the urgent need to design effective interventions in pregnancy to prevent excessive gestational weight gain and postpartum weight retention [69]. To date, no intervention has been published that has targeted behavioral changes in relation to eating and physical activity as well as changes in psychological factors such as motivation, confidence, mood, and body image concerns, with the aim of preventing excessive gestational weight gain and 12-month postpartum weight retention. Our pregnancy HC Intervention is, therefore, novel and innovative. Moreover, it is unique in its application of health coaching principles and strategies to the life stage of pregnancy, that have been successful in instigating behavioral changes, in relation to chronic diseases including obesity, at other life stages. Our HC Intervention has been designed to be easily adopted by health professionals who work with pregnant women, such as obstetricians, midwives, GPs, and health psychologists. We additionally hypothesize that this intervention will provide a cost-effective approach to prevention of excessive gestational weight gain.

We expect the information obtained from this trial will be used to inform gestational and postpartum health practices and develop public health policies in relation to the prevention of excessive weight gain in pregnancy and weight retention post birth.

## Competing interests

The authors declare that they have no competing interests.

## Authors' contributions

Together, HS, MM, JM, BK wrote and designed the study subsequently funded by the National Health and Medical Research Council, and modified it for publication. CH will oversee the economic evaluation of the project. SH will contribute to the interpretation and publication of findings. MB and GT will provide departmental support and will advise on obstetric issues that arise. DP will provide organizational oversight at Eastern Health and advise on specific midwifery issues. JG will supervise the practice of the Health Coaches. JL is the Health Coach appointed to the project. LB is the research assistant appointed to manage the collection of data. All authors read and approved the final manuscript.

## Pre-publication history

The pre-publication history for this paper can be accessed here:

http://www.biomedcentral.com/1471-2458/12/78/prepub
